# Clinical outcomes and patient perspectives of Dental Monitoring® GoLive® with Invisalign®—a retrospective cohort study

**DOI:** 10.1186/s40510-020-00316-6

**Published:** 2020-06-15

**Authors:** Ismaeel Hansa, Steven J. Semaan, Nikhilesh R. Vaid

**Affiliations:** 1 Durban, South Africa; 2 Department of Orthodontics, European University College, Dubai, UAE; 3 Department of Orthodontics, University of Nevada, Las Vegas, Las Vegas, USA; 4 Gold Coast, Australia

**Keywords:** Teledentistry, Teleorthodontics, Dental Monitoring®, Remote monitoring, Digital technology

## Abstract

**Background:**

The aims of the study were to compare the effects of Invisalign® with and without Dental Monitoring® (DM) GoLive® on the following parameters: treatment duration, number of appointments, number of refinements, total number of refinement aligners, and time to initial refinement. The patients’ perspectives on Dental Monitoring® were also evaluated using an online questionnaire. A sample of 155 consecutively treated Invisalign® patients (67 control, 88 DM) fit the inclusion and exclusion criteria.

**Results:**

The two groups were homogeneous (*P* > .05) for age, gender, angle classification, Little’s Irregularity Index, and number of initial aligners. The DM group had significantly fewer office visits compared to the control (7.56 vs 9.82; *P* < .001). There were no significant differences between the DM and control groups respectively pertaining to treatment duration (14.58 vs 13.91), number of refinements (1.00 vs 0.79), number of refinement aligners (19.91 vs 19.85), and time to first refinement (9.46 vs 9.97). Questionnaire results showed that 68.8% (44 respondents) indicate that DM scans were “easy” or “very easy” to perform while 16 responders (25%) found it “difficult” or “very difficult.” 71.9% (46 responders) were “satisfied or very satisfied” with the level of communication with the orthodontist using DM and 16% (10 responders) were “dissatisfied” or “very dissatisfied.” The mean duration observed by patients to take a scan was 5.16 ± 3.6 min. Eighty-eight percent (56 responders) of patients prefer few office visits as possible, while 12% (8 responders) would actually prefer additional office visits. Overall, the mean satisfaction of patients with DM was 4.25 on the 5-point Likert scale.

**Conclusion:**

The DM group had a significantly reduced number of appointments (7.56) compared with the control group (9.82) (a reduction of 23%) over the treatment duration. There were no significant differences between the two groups in treatment duration, number of refinements, number of refinement aligners, or time to 1st refinement. Overall, DM was well received by patients. However, there was a small percentage (usually less than 15%) that was generally unsatisfied with DM in varying aspects and preferred more frequent, traditional office visits.

## Background

Teleorthodontics and remote monitoring are an imminent reality which permits orthodontists to proactively monitor their patients with virtual examinations to supplement chairside appointments [1, 2]. Although tainted with negative connotations associated with the direct to consumer business model [3, 4], there are some tremendous advantages of remote monitoring to the practice of orthodontics [5]. First and foremost is reducing (not eliminating) the need for in-office visits. This is beneficial, both for the orthodontist and the patient, in an increasingly time-conscious world. The orthodontist can improve treatment and chairside efficiency, while the patients avoid the extra financial and time costs of traveling to the practice [6, 7]. Areas with limited access to orthodontic care can benefit greatly from remote monitoring as regular appointments become unfeasible [8, 9]. Early interception of developing problems such as poor oral hygiene, non-tracking aligners, broken appliances, or poor compliance may also help reduce treatment times [10, 11].

Remote monitoring may not be well suited to traditional orthodontics, as frequent chairside activations are required. On the other hand, customized appliances such as clear aligner treatment may take full advantage of remote monitoring due to the preprogrammed tooth movement. Consequently, appointments requiring simple evaluation may be eliminated [7].

Dental Monitoring® (DM) (Paris, France) [12] is a software that allows patients to accurately capture their dentition using a smartphone and patented cheek retractors. Their GoLive® option is specifically targeted at clear aligner treatment. Instead of conventional planned aligner changes, the patients receive a weekly “GO” or “NO-GO” notification which paired with the orthodontists customized pre-recorded instructions, indicates whether they should proceed to the next aligner or remain in the current one. The orthodontist is informed when a NO-GO notification is sent, and individual teeth tracking issues, poor oral hygiene, or broken attachments can be identified. The orthodontist can override a NO-GO if desired.

Hansa et al. [2] found a generally positive patient perception and experience using DM, with the most common complaint being unable to take DM scans correctly. Common benefits mentioned by patients included better communication with the orthodontist, reduced number of office visits, and increased convenience. Morris et al. [13], Moylan et al. [14], and Kuriakose et al. [15] have all studied and confirmed the clinical accuracy of DM in measuring and tracking tooth movement.

There are currently no published studies evaluating the clinical implications and performance of Dental Monitoring® GoLive® with Invisalign® (Align Technology, Santa Clara, Calif). The aims of the present investigation were to compare the effects of Invisalign® with and without DM on the following parameters: treatment duration, number of appointments, number of refinements, total number of refinement aligners, and time to initial refinement and to further evaluate patient perspectives of DM. The null hypothesis was that there is no difference between the two groups with regard to the aforementioned parameters.

## Methods

Approval for this retrospective study was granted by the institutional review board at the European University College. Treatment was provided by an experienced (Red Diamond) Invisalign® provider in Gold Coast, Australia. Patients were given the choice of using DM or not after being informed about its pros and cons and were treated at no additional cost compared to the control group. All DM patients were trained in the use of the DM app at the aligner delivery appointment.

The sample size was calculated based on an alpha significance level of 5% and 95% power in detecting a difference of 3.52 (± 3.71) number of appointments based on the results of Hansa et al. [16]. The results showed that a minimum of 27 patients was needed for each group. However, our other objectives have unknown or smaller effect sizes, which necessitated a larger sample size in order to obtain adequate power in determining true differences. Hence, the study consisted of a total of 215 consecutively treated patients using clear aligner therapy (94 control; 121 DM). One hundred fifty-five patients (67 control, 88 DM) fit the following inclusion criteria: (1) between 30 and 65 initial aligners, (2) non-extraction treatment, (3) a complete permanent dentition anterior to and including first molars, and (4) treatment with the default amounts of tooth movement in each aligner stage. Patients were excluded based on (1) treatment in combination with fixed appliances or other auxiliary appliances, (2) treatment with Invisalign® molar distalization or mandibular advancement in class II patients, (3) orthognathic surgery, (4) the presence of dental prostheses, (5) the use of any DM option other than GoLive®, and (6) the use of any aligner systems other than Invisalign®.

The pre-treatment data recorded were age, gender, Little’s Irregularity Index, angle classification, and number of initial aligners. In addition, each patient was observed for treatment duration (in months), number of refinements, number refinement aligners, time to first refinement, and number of appointments.

An online questionnaire was given only to the DM group during treatment to assess the patients’ perspective on the ease of use and satisfaction on a 5-point Likert scale. They were also requested to indicate if they would prefer more physical visits, the duration it takes to complete a DM scan, and how often are the scans rejected and required retaking. We also asked about the patients’ travel time to the office. Sixty-four patients (73%) responded to the questionnaire.

### Statistical analysis

The data was compiled using Microsoft Excel (Microsoft, Redmond, Wash) and thereafter analyzed with SPSS® Statistics (Version 25, Chicago, Illinois, USA). *P* values of less than 0.05 were considered statistically significant. Interval data were tested for normal intra-group distribution using the Shapiro-Wilk test. Independent *t* tests were used for normally distributed data, and Mann-Whitney *U* tests were used if the data was not normally distributed. Chi-square tests were used for nominal data testing.

## Results

The two groups were tested for pre-treatment differences in sample size, age, gender, angle classification, Little’s Irregularity Index, and number of initial aligners. The two groups were well matched with no significant differences found using two-tailed independent *t* tests for interval data and chi-square tests for nominal data (Table 1).

**Table 1:** Pretreatment variables

	*n*	Age (years)	Gender (M:F)	Angle class. (I:II:III)	Mn irreg. index (mm)	No. initial aligners
DM	88	25.3 ± 11.1	63:25	40:45:3	5.9 ± 3.0	42.2 ± 7.7
Control	67	25.4 ± 10.1	50:17	29:36:2	5.9 ± 2.6	44.7 ± 12.3
*P* signif.		0.976	0.177	0.110	0.963	0.15

The only significant difference between the two groups was the mean number of appointments. The DM group had 2.26 (23%) fewer visits compared to the control (7.56 vs 9.82; *P* < .001) (Table 2). There were no significant differences between the DM and control groups respectively pertaining to treatment duration (14.58 vs 13.91), no. of refinements (1.00 vs 0.79), number of refinement aligners (19.91 vs 19.85), and time to first refinement (9.46 vs 9.97).

**Table 2:** Results

	DM		Control			
	Mean	SD	Mean	SD	Mean dif.	*P* signif.
TX. duration (months)	14.58	3.30	13.91	5.28	-0.67	.335
No. of refinements	1.00	0.77	0.79	1.01	-0.21	.146
No. of refinement aligners	19.91	12.10	19.85	14.99	-0.06	.983
Time to 1st refinement	9.46	3.78	9.97	4.65	0.51	.567
No. appointments	7.56	2.79	9.82	3.68	2.26	< .001

**Fig. 1:**
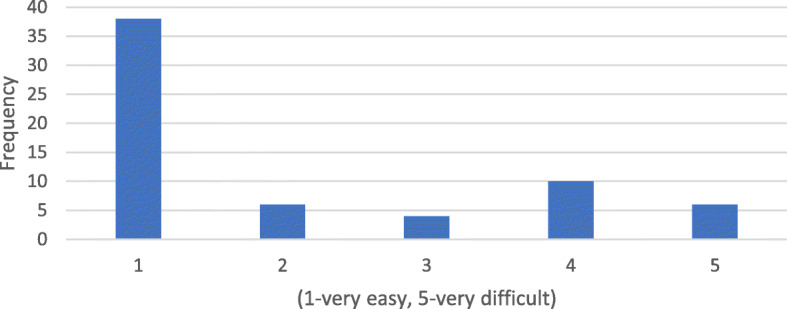
How difficult is it to take scans using DM?

**Fig. 2:**
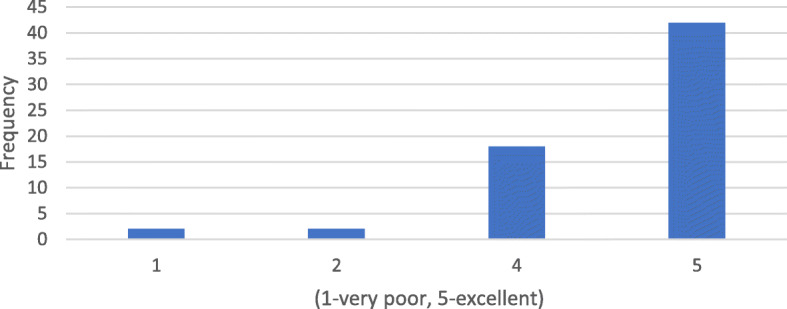
How would you rate your ability to perform weekly scans regularly and on time?

**Fig. 3:**
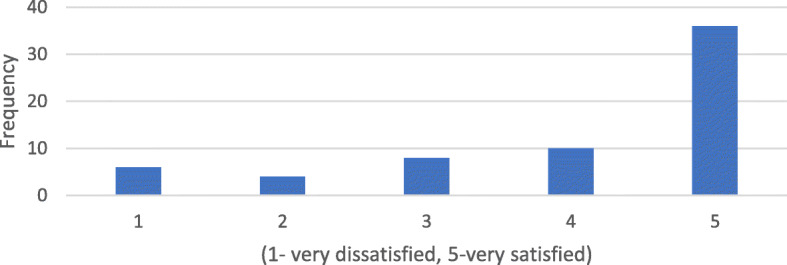
Are you satisfied with the level of communication with the orthodontist using DM?

Questionnaire results showed that 68.8% (44 respondents) indicate that DM scans were “easy” or “very easy” to perform. Sixteen responders (25%) found it “difficult” or “very difficult” to take DM scans correctly. The mean difficulty was 2.06 on a 5-point Likert scale (Fig. 1). When asked to rate their ability to take scans regularly and on time, which is essential for successful treatment with DM, the mean Likert scale rating was 4.5 out of 5. However, 9% (4 responders) indicated that they would be “poor” or “very poor” at taking scans regularly (Fig. 2). 71.9% (46 responders) were “satisfied or very satisfied” with the level of communication with the orthodontist using DM. Although 16% (10 responders) were “dissatisfied” or “very dissatisfied,” a mean score of 4.03 was obtained (Fig. 3).

**Fig. 4:**
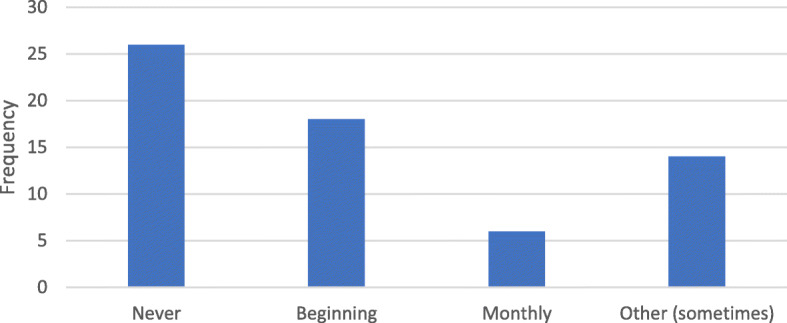
How often are your scans rejected and require retaking?

**Fig. 5:**
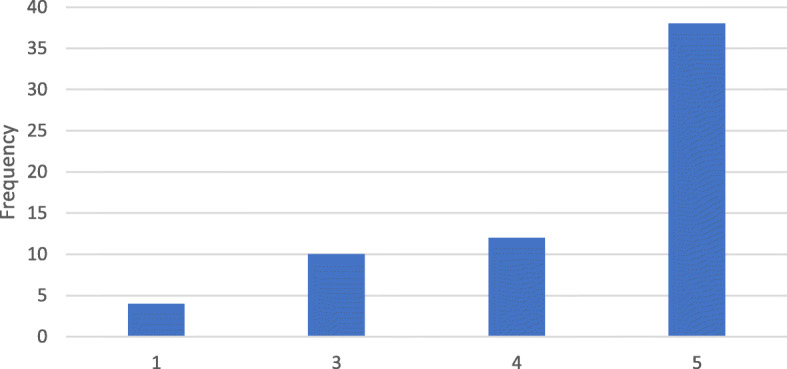
Overall satisfaction

**Fig. 6:**
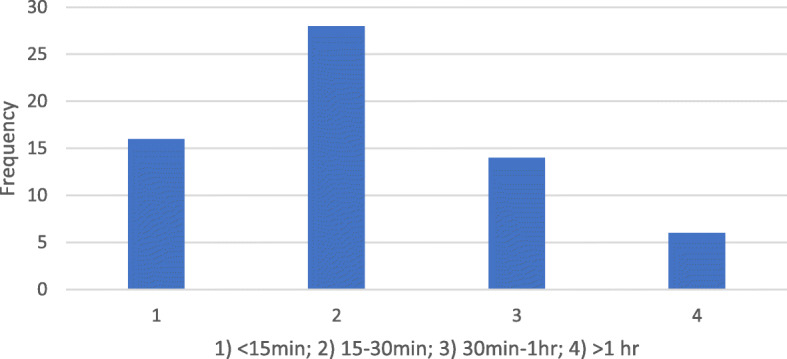
Travel time to office

When asked about how frequently scans were rejected and required retaking, 41% (26 responders) indicated that no scans were ever rejected; 28% (18 responders) indicated that scans required retaking during the early stages of treatment and with more experience scans were no longer rejected. Twenty-two percent (14 responders) indicated that scans were rejected occasionally, while 9% (6 responders) indicated that scans were rejected at least once a month. The mean duration taken by patients to take a scan was 5.16 ± 3.6 min. Seventy-five percent (48 responders) took less than or equal to 5 min. However, the duration ranged from 2 to 17 min (Fig. 4). Eighty-eight percent (56 responders) of patients prefer few office visits as possible, while 12% (8 responders) would actually prefer additional office visits. Overall, the mean satisfaction of patients with DM was 4.25 on the 5-point Likert scale. 78.1% were satisfied or very satisfied, while 6% (4 responders) were very dissatisfied with DM (Fig. 5). The travel time of the majority of patients (44%) was between 15 and 30 min. Twenty-five percent of patients traveled less than 15 min, 22% took between 30 min and 1 h, and 9% traveled greater than 1 h (Fig. 6).

## Discussion

A reduction of the number of appointments by 2.26 visits (23%) over the treatment duration in the DM group was both statistically significant and clinically relevant, thus rejecting the null hypothesis. The reduction in appointment number using DM also confirms the results of the pilot study by Hansa et al. [2] and an unpublished thesis [16]. A reduction in appointments reduces social costs in general. Specifically, it benefits the orthodontist in terms of efficiency and the patient by increasing convenience and reducing time and travel costs. The decision to embrace this technology will be based on the individual office’s demographics, costs, scheduling, and protocols.

There were no statistically significant differences between the two groups for treatment duration, number of refinements, number of refinement aligners, or time to first refinements. These results suggest that the idea of using DM for early interception of developing problems for reducing treatment times is misplaced, as both 1st refinements occurred around the 9th month of treatment, and treatment duration actually favored the control group (albeit not statistically significant). It should be noted that both groups used only elastics in the treatment of sagittal discrepancies, as including other modalities such as molar distalization protocols, mandibular advancement aligners, or auxiliaries may have introduced confounding factors. Crowding in both groups was resolved by a combination of inter-proximal reduction (IPR) and proclination. The amount of IPR performed or the amount of proclination accepted was decided on a patient-to-patient basis, but there were no set protocols. The unpublished thesis by Hansa [16] also showed that overall refinement needed was similar between the two groups. However, they found that the time to refinement was reduced in the DM group, which was not found in this study. This difference may be due to the individual office’s varying refinement protocols when minor problems are detected.

The questionnaire results suggest patients were overall satisfied with DM and the level of communication, with a mean rating of 4.25 and 4.03, respectively, on a 5-point Likert scale. The DM app allows for direct message communication with the orthodontic office, which mitigates the communication problems that may occur with less frequent office visits.

While the DM app was easy to use for a majority of patients (68.8%) in the present study, with a mean difficulty of 2.06; 25% of patients found scans difficult to be performed. According to patients, it takes on average 5.16 ± 3 min per scan, which is important for prospective patients to note prior to accepting treatment with DM. The amount of time taken by patients ranged from 2 to 17 min and has a large variability. DM has attempted to help patients by introducing the Scanbox [12], which supposedly makes taking scans quicker, easier, and more reliable. The Scanbox is a newly released optional auxiliary that has an added cost for the orthodontist. The patients in this study did not use the Scanbox, and thus, the ease of use of DM may differ if the patients used the Scanbox.

Although the majority of patients (88%) would prefer as few office visits as possible, surprisingly, a significant percentage of patients (12%) actually would prefer to have more office visits. Reducing the number of face-to-face appointments may diminish the rapport between doctor and patient. This traditional relationship may be reduced or lost, and with that, possibly confidence as well [17]. Dunbar et al. [18] reported in a pilot study that 70% of subjects felt that the face-to-face aspect was extremely important, and the majority preferred this over the exclusive use of teleorthodontic technology. It was interesting to note that 25% of the DM group’s travel time to the office was less than 15 min, and only 9% was greater than 1 h. It would seem then, that in this study at least, patients did not choose to utilize DM based primarily on their commute length to the office.

While DM appears to be well received by patients in general, there was a small percentage (usually less than 15%) that was unsatisfied with DM and preferred more traditional office visits. It could be hypothesized that the more technologically adept patients may have an easier time using DM. However, patients will need to decide whether the weekly or bimonthly at-home scans are a greater convenience than office visits according to the office’s protocol.

The key to remote monitoring seems to be in balancing the benefits of in-office visits and direct patient-doctor relationships with the convenience and perhaps reduced costs of remote monitoring, based on an individual patient-to-patient, and office-to-office basis.

This is one of the first studies on the real-world performance of remote monitoring, and some limitations are present. Foremost is the intrinsic bias of a retrospective, non-randomized study. The second is the question of external validity. All patients were from a single practice in Australia and thus may not represent the demographics in other parts of the world.

## Conclusions

- The DM group had a significantly reduced number of appointments (7.56) compared with the control group (9.82).
- There were no significant differences between the two groups in treatment duration, number of refinements, number of refinement aligners, or time to 1st refinement.
- DM appears to be generally well received by patients. There was however a small percentage (usually less than 15%) that was generally unsatisfied with DM in various aspects and may prefer more frequent, traditional office visits.
- Overall, 78% were satisfied or very satisfied with DM, 16% were content, while 6% were very dissatisfied.

## Data Availability

The datasets used and/or analyzed during the current study are available from the corresponding author on reasonable request.

## Abbreviations

DM: Dental Monitoring®
IPR: Inter-proximal reduction
